# Magnetic Carbon Nanofiber Mats for Prospective Single Photon Avalanche Diode (SPAD) Sensing Applications

**DOI:** 10.3390/s21237873

**Published:** 2021-11-26

**Authors:** Marah Trabelsi, Al Mamun, Michaela Klöcker, Imane Moulefera, Anton Pljonkin, Khaled Elleuch, Lilia Sabantina

**Affiliations:** 1 Ecole Nationale d’Ingénieurs de Sfax, Laboratory LGME, University of Sfax, Sfax 3038, Tunisia; marah.trabelsi@enis.tn (M.T.); khaled.elleuch@enis.tn (K.E.); 2 Junior Research Group “Nanomaterials”, Faculty of Engineering and Mathematics, Bielefeld University of Applied Sciences, 33619 Bielefeld, Germany; al.mamun@fh-bielefeld.de; 3 Faculty of Engineering and Mathematics, Bielefeld University of Applied Sciences, 33619 Bielefeld, Germany; michaela.kloecker@fh-bielefeld.de; 4 L.M.A.E. Laboratory, Department of Process Engineering, Faculty of Science and Technology, University of Mustapha Stambouli, Mascara 29000, Algeria; imanemoulefera@yahoo.fr; 5 Institute of Computer Technology and Information Security, Southern Federal University (SFedU), 347900 Taganrog, Russia; pljonkin@mail.ru

**Keywords:** carbon nanofibers, carbonization, magnetic nanoparticles, magnetite, PAN

## Abstract

Electrospinning enables simple and cost-effective production of magnetic nanofibers by adding nanoparticles to a polymer solution. In order to increase the electrical conductivity of such nanofibers, the carbonization process is crucial. In this study, the chemical and morphological properties of magnetic nanofiber mats prepared from polyacrylonitrile (PAN)/magnetite were investigated. In our previous studies, PAN/magnetite nanofiber mats were carbonized at 500 °C, 600 °C, and 800 °C. Here, PAN/magnetite nanofiber mats were carbonized at 1000 °C. The surface morphology of these PAN/magnetite nanofiber mats is not significantly different from nanofiber mats thermally treated at 800 °C and have remained relatively flexible at 1000 °C, which can be advantageous for various application fields. The addition of nanoparticles increased the average fiber diameter compared to pure PAN nanofiber mats and improved the dimensional stability during thermal processes. The high conductivity, the high magnetization properties, as well as shielding against electromagnetic interference of such carbonized nanofibers can be proposed for use in single photon avalanche diode (SPAD), where these properties are advantageous.

## 1. Introduction

**Figure 1:**
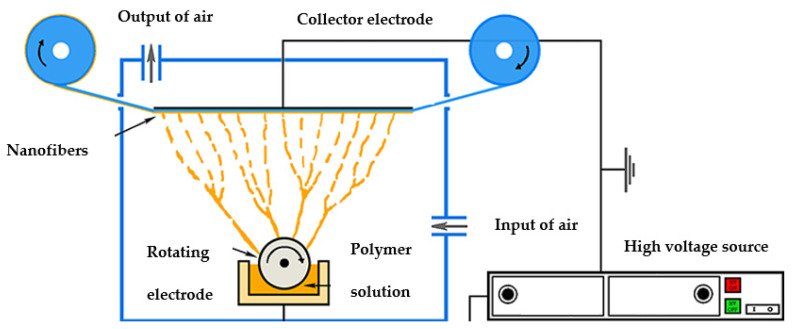
Schematic of the needle-less electrospinning setup. Adapted with permission from reference [11], Sasithorn et al., 2016, originally published under a CC-BY 3.0 license.

Magnetic nanofibers are produced by introducing magnetic nanoparticles into a polymer solution using an electrospinning process, which is simple and cost-effective [1,2,3]. Due to their high surface-to-volume ratio and high porosity, nanofibers have excellent sensitivity and high sensing performance, which makes them attractive for sensing applications such as detection of various gases, strain sensors, etc., [4,5]. Electrospinning is a very efficient and versatile method for manufacturing nanofibers on a large scale and at low cost [6,7,8]. In general, all electrospinning techniques are based on the following procedure: polymer is dissolved in a solvent and this polymer solution or melt is introduced into an electric field. By applying a high voltage between two electrodes, Taylor cons are simultaneously formed and result in ultrathin nanoscale fibers, which are deposited on the substrate [9,10]. In general, needle-based and needle-free electrospinning techniques are often used. With needle-free electrospinning technology, on which the Nanospider^®^ electrospinning machine (Elmarco s.r.o., Liberec, Czech Republic) is based, it is possible to produce relatively large quantities of nanofibers in the same quality. The schematic layout of the Nanospider^®^ Lab machine is shown in Figure 1.

Polyacrylonitrile (PAN) polymer is often used to prepare nanofiber mats [12] as it exhibits water-resistant properties and is applicable in many defined areas requiring this property, such as water purification, filtration, or medical areas [13,14]. In addition, PAN is often used for the production of carbon nanofibers, as it is known to have a high carbon yield [15,16]. Carbon nanofibers exhibit high electrical conductivity, low density, as well as high specific strength. Moreover, carbon nanofiber mats are easy to handle and overcome all major challenges in dispersion, processing, and handling compared to other carbon nanomaterials such as carbon nanotubes [17,18,19,20].

Magnetic nanofibers are of great interest for basic research of their magnetic properties as well as for possible applications in spintronics, neuromorphic computing and data transfer [21,22,23]. Secure data transfer is of great interest nowadays and requires effective strategies [24,25]. For some kinds of applications, nanofiber mats with agglomeration and beads are advantageous because magnetic nanoparticles collect in beads and thus form a network with nodes [2]. Typically, the formation of beads in nanofiber mats is not desired, and various strategies are proposed to avoid the formation of beads, such as the use of surfactants, mechanical mixing or surface modification [26]. Changing the viscosity of the electrospinning solution, changing the amount of polymers, changing the distance between the electrode and the substrate on which the nanofibers accumulate to get the nanofiber mats morphology from nanofiber mats to membrane, mixing the polymer solution by ultrasound etc., are just some of the many other ways to change the nanofiber mats morphology mentioned here [27,28,29,30].

The nanofiber mats prepared by electrospinning and carbon nanofiber mats are widely used in the fields of filtration, separation, sensors, catalyst support, energy storage, biomedicine, shielding from radiation, etc., [31,32,33,34,35,36,37]. The application of carbon nanofibers in the field of sensing elements is based on their electronic transport properties, which are modulated by the physical-chemical interaction of the sensing element [38]. One-dimensional (1D) nanomaterials, such as nanofibers, contain shortened paths for electron transfer and favor the penetration of electrolyte along the longitudinal axis of nanofibers, which results in improved sensor performance [39].

Many chemoresistive detectors are prepared using electrospinning methods to obtain easy-to-produce micrometer-sized detectors with high sensitivity, increased detection rates, stability, and cross sensitivity [36,40,41]. Ali et al. presented the development of an immunosensor based on mesoporous zinc oxide nanofibers (ZnO-NFs) for breast cancer screening [42]. Chowdhury et al. developed an impedimetric biosensor using polyaniline (PANI) nanofibers that were deposited on a gold (Au) electrode using the cyclic voltammetry technique for the detection of Escherichia coli O157:H7 bacteria [43]. Zhang et al. used graphene oxide (GO) nanofibers decorated with nickel oxide (NiO) for the electrochemical detection of glucose [44]. Luo et al. reported a lateral flow immunosensor based on electrospun cellulose nitrate nanofibers and magnetic nanoparticles (γ-Fe_2_O_3_) to detect *E. coli* O_157_:H_7_ colonies [45]. Moreover, Bahrami et al. manufactured a sensitive electrochemical sensor from PVA with iron salts (FeCl_2_:FeCl_3_) as magnetic fibers combined with graphite powder and paraffin oil for morphine analysis in biological samples [46]. In addition, magnetic nanofibers are widely used in the field of wires. An electrospun polycarbonate urethane polymer (PCU) with nickel (Ni) was prepared, and its nanostructured wires exhibited high longitudinal elastomagnetic strain and high transverse deflection in response to magnetic field stimuli [47]. The magnetic carbon nanofibers can be used in wastewater treatment. Li et al. successfully synthesized the Fe_3_O_4_/PAN-modified magnetic carbon nanofibers (MNFs) by electrospinning and produced horseradish peroxidase-(H-MNFs), which achieved a phenol removal efficiency of 85% the first time and 52% after recycling five times [48]. On the other hand, magnetic nanofibers with core (Fe_3_O_4_ nanoparticle suspension)/sheath (polyethylene terephthalate) structure were successfully fabricated by electrospinning [49]. The obtained magnetic nanofibers showed superparamagnetic behavior at room temperature as well as favorable mechanical properties, and this leads to many application areas, such as sensors and/or smart bulletproof vests where these magnetic nanofibers can be used [50].

The fact that conductivity increases with increasing carbonization temperature was confirmed in several studies [51,52]. Carbon nanofibers belong to an important class of graphitic materials and are similar in structure in many ways to crystalline three-dimensional graphite. During oxidative stabilization and carbonization processes, the aromatic groups or the groups with ring structure due to the cyclization or crosslinking of the precursor chains lead to aromatization. These processes require the formation of carbon nanofibers with uniform carbon structure and promote electronic mobility, which results in high electrical conductivity [53].

The defined properties and the use of magnetic nanofiber mats were discussed in several studies. In the study by Zuo et al., porous magnetic carbon nanofibers (P-CNF/Fe) were fabricated and absorption of electromagnetic waves was investigated. The minimum reflection coefficient reached −44.86 dB at 4.42 GHz. Moreover, the largest effective absorption bandwidth (EAB) was in the frequency range from 12.96 to 16.24 GHz and reached the value of 3.28, which indicates the approach of such P-CNF/Fe in EM wave attenuation [54]. Fe_3_O_4_/carbon composite nanofibers prepared from polyacrylonitrile (PAN)/acetylacetone iron (AAI) and carbonized at 800 °C were evaluated in the study by Zhang et al. for microwave absorption. It was found that adding Fe_3_O_4_ particles leads to an improvement in electrical conductivity [55]. Bayat et al. investigated the effectiveness of electromagnetic interference (EMI) shielding effectiveness (SE) by multifunctional Fe_3_O_4_/carbon nanofibers. The maximum EMI SE of 67.9 dB was obtained [56].

In our previous study by Döpke et al., PAN/magnetite nanofiber mats were prepared and the magnetic properties were investigated [2]. Here, networks of magnetic nanofibers with beads that can be used to transport data in the form of domain walls moving along them and combined with objects that could be used to store data and transport [2,3,4,5,6,7,8,9,10,11,12,13,14,15,16,17,18,19,20,21,22,23,24,25,26,27,28,29,30,31,32,33,34,35,36,37,38,39,40,41,42,43,44,45,46,47,48,49,50,51,52,53,54,55,56,57] are of interest. The use of such fibers is attractive not only for sensing applications but also for other technologies where these properties are advantageous. In a previous study by Fokin et al. [58], magnetic nanofibers were also oxidatively stabilized and carbonized at 500 °C and 800 °C in order to increase the electrical conductivity and enhance stronger contraction and networking of nanofibers to each other, being supportive of magnetic data transmission and other application areas where these properties are beneficial. Usually, when considering scientific literature, different strategies are proposed to avoid agglomeration and beads in nanofibers. In our study and other aforementioned studies, the beads are advantageous because it is assumed that magnetic nanoparticles accumulate there and are crucial for the electrical, magnetic, mechanical, and other properties. In our previous study by Trabelsi et al. [21], PAN/magnetite nanofiber mats were prepared and incipiently carbonized at 500 °C. It was found that visual observation by confocal laser scanning microscopy (CLSM) or scanning electron microscopy (SEM) could not clearly detect agglomerations in the nanofibers. However, using energy dispersive X spectroscopy (EDS), nanofibers can be easily analyzed to detect agglomerations of magnetic nanoparticles without the use of costly techniques and preparation time such as TEM [21]. The magnetic properties of PAN/magnetite nanofiber mats were investigated on the nanoscale by Weiss and Ehrmann using magnetic force microscopy (MFM) [59], showing less clear differentiation between magnetic and polymeric parts than EDS. The carbonization of PAN/magnetite nanofiber mats at 500 °C, 600 °C, 800 °C, and 1000 °C was carried out by Trabelsi et al. Based on the study mentioned, this study was extended and supplemented [60].

Nanofiber mats and nanofiber mats with magnetic particles can be used in many applications due to their excellent properties such as super capacitance, excellent mechanical properties, and shielding against electromagnetic interference [61,62]. These properties offer a potential application of magnetic carbon nanofiber mats for use in single photon avalanche diodes (SPADs), where high conductivity and high magnetization properties as well as shielding against electromagnetic interference are important to improve the construction design of such sensors.

The SPAD arrays can be considered as solid-state detectors providing imaging capabilities at the single photon level and are used in areas of data and telecommunication security, quantum key distribution applications, advanced driver assistance systems, fluorescence detection, remote-sensing technology, biophotonics, and robotics [63,64,65,66,67,68,69,70]. Advantages of SPADs include unprecedented photon counting and time-resolved performance, and therefore they can be used when deep sub-nanosecond timing performance is required [63,64,68]. On the technological side, the development of high-performance and low-noise SPADs is challenging because many aspects are considered, and compromises have to be made to balance sensitivity against noise and speed against fill factor [70].

The new application areas for optical quantum information and quantum key distribution (QKD) require the rapid developments in detector performance [71,72,73]. There is a great need for new device concepts that can be used practically for optical quantum information technologies, because the conventional photon counting detectors cannot technically meet all the requirements [74]. New detector technologies are constantly evolving and are highly relevant for many applications such as optical quantum information technologies [75]. Semiconductor detectors, such as SPADs, are used in the fields of quantum optics and quantum information transfer. Currently, some challenges are still observed in the area of secure data transfer. The performance of SPADs is not yet mature enough to ensure secure quantum information such as the distribution of quantum keys over long distances in optical fibers [75].

Most detectors include a probability of recording false counts such as dark current pulses (DCPs), dark counts, or dark noise [76]. These effects are caused by the material properties of the detector, bias conditions, or susceptibility to external noise. Protection from these negative effects will significantly improve the performance of SPADs.

**Figure 2:**
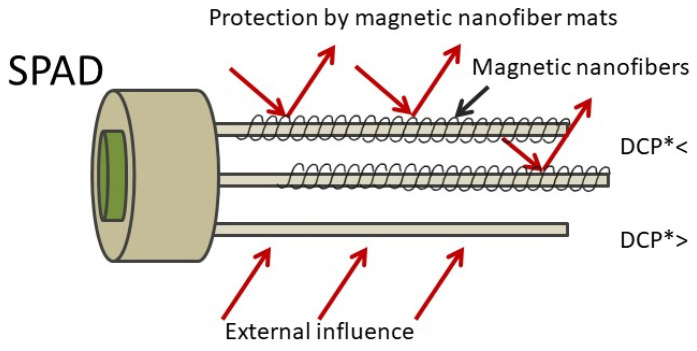
Schematic illustration of the novel single-photon avalanche diode (SPAD) design and the potential use of magnetic nanofiber mats to reduce external influences. The abbreviation (DCP*) means dark current pulses.

In Figure 2, the novel construction design for the SPAD is presented and the possible use of magnetic nanofibers as a protection against external noise by electromagnetic shielding is proposed in this study and serves as a basis for further development. The nanofibers are expected to reduce dark current pulses (DCPs) generated by external noise. The DCP level is directly related to quantum errors (QBER), and the lower the DCP, the lower the QBER, which is beneficial for the speed of signal transmission.

An avalanche photodiode SPAD is based on Geiger-mode principle, where the electric field of the junction is so strong that even a single charge carrier such as electron or hole causes an avalanche. To restore the original bias state, a passive or active quenching circuit is needed [77].

In the Wang et al. study, electric-field-drive single photon avalanche diode with barrier enhancement was investigated for fluorescence detection [78]. The study by Ejdehakosh et al. deals with the development of new optical random number generator and a simulation with the use of post-layout in 180 nm standard CMOS (complementary metal oxide semiconductor) technology. The circuit was designed using three parallel SPADs with the aim of detecting photons. To reduce the dark count rate and post-pulsing, one of the three SPADs was biased in Geiger-mode while the other two SPADs were in hold off mode. Here, the SPAD circuit model served to verify the functionality of the circuit [79]. The effect of the electric field on primary dark pulses in SPADs for advanced radiation detection applications was investigated in the study by Lim et al. [80]. The arrays of SPADs include many positive features, such as compactness, high detection efficiency, low operating voltage, magnetic field resistance, rapid time response, and can therefore replace conventional photomultiplier tubes (PMTs) in many radiation detection applications [80].

This study deals with electrospun magnetic nanofiber mats of polyacrylonitrile (PAN) and magnetite nanoparticles (Fe_3_O_4_) using the low toxic solvent dimethyl sulfoxide (DMSO). The different nanofiber morphologies of the nanofiber mats that were oxidatively stabilized and carbonized at 500 °C, 600 °C, 800 °C, and 1000 °C were investigated using Confocal laser Scanning Microscope (CLSM), Atomic Force Microscope (AFM), and Scanning Electron Microscope/Energy Dispersive X-Ray Spectroscopy (SEM/EDS), Fourier-transform infrared spectroscopy (FTIR) and are discussed below. The focus of this study is the carbonization of PAN/magnetite nanofiber mats at higher temperatures of up to 1000 °C and the characterization of the surface morphology of the resulting nanofiber mats. The novelty of this study is that, according to our research and to the best of our knowledge, there is no comparable study describing exactly this composition of PAN/magnetic nanofibers, such as 14% PAN and 20% magnetite, using low-toxic solvent DMSO, produced by needle-free electrospinning machine Nanospider^®^ Lab and carbonization at 1000 °C, and focusing on morphology of these nanofiber mats.

The properties of such magnetic carbon nanofiber mats are promising for a novel construction design of SPAD where these properties are advantageous. The high conductivity, the excellent mechanical properties as well as the large magnetization and shielding against electromagnetic interference of magnetic carbon nanofibers can reduce noise in (SPAD) sensors and increase the speed of transmission of signals, and the present work aims to provide the basis for this application.

## 2. Materials and Methods

The polymer spinning solution contains 14 wt.% polyacrylonitrile (X-PAN, Dralon, Dormagen, Germany) dissolved in low-toxic solvent dimethyl sulfoxide (DMSO) (min. 99.9%, purchased from S3 chemicals, Bad Oeynhausen, Germany) and 20% magnetic nanoparticles Fe_3_O_4_ (magnetite, 50–100 nm diameter, Merck KGaA, Darmstadt, Germany). The polymer solution was stirred in a magnetic stirrer for 2 h and before electrospinning additionally treated in an ultrasonic bath for 40 min at 35 °C with a frequency of 37 kHz. The needleless electrospinning machine Nanospider Lab (Elmarco, Liberec, Czech Republic) was applied.

The nanofibers were produced at the following spinning parameters: High voltage 80 kV, electrode-substrate distance 240 mm, bottom-substrate distance 50 mm, nozzle diameter 0.9 mm, carriage speed 150 mm/s. The temperature in the chamber was 22 °C and relative humidity was 33%. The duration of electrospinning amounted to 15 min.

In our previous studies, it was experimentally found that the polymer amount between 14% and 16% of PAN dissolved in DMSO and the electrospinning parameters used in this study are most suitable for the production of homogeneous nanofibers using the electrospinning machine Nanospider^®^ Lab [1,2,4,21,27,58,81].

Oxidative stabilization of the nanofiber mats was carried out in an oven (Nabertherm, Lilienthal, Germany). The typical stabilization temperature at 280 °C with a heating rate of 1 K/min was applied. Afterwards, an isothermal treatment was carried out at this final temperature for 1 h. Carbonization occurred in a furnace (Carbolite Gero, Neuhausen, Germany). The carbonization temperatures were set to 500 °C, 600 °C, 800 °C, and 1000 °C at a heating rate of 10 K/min in a nitrogen flow of 150 mL/min (STP). After reaching the final temperature, isothermal treatment was performed for 1 h.

Nanofiber diameters were examined based on analysis of SEM micrographs using ImageJ (software version 1.53e, 2021, National Institutes of Health, Bethesda, MD, USA). For the determination of the nanofiber diameter distribution, 100 fibers were measured.

Optical investigations were made with a FlexAFM Axiom (Nanosurf, Liestal, Switzerland) and a confocal laser scanning microscope (CLSM), VK-8710 (Keyence). A Zeiss 1450VPSE scanning electron microscope (SEM) and energy-dispersive X-ray spectroscopy (EDS) were used for more detailed investigations. For Fourier-transform infrared (FTIR) spectroscopy, an Excalibur 3100 (Varian, Inc., Palo Alto, CA, USA) was used. The spectral range from 4000 cm^−1^ to 700 cm^−1^ was set and 32 scans were averaged in each case and atmospheric noise was corrected.

## 3. Results

**Figure 3:**
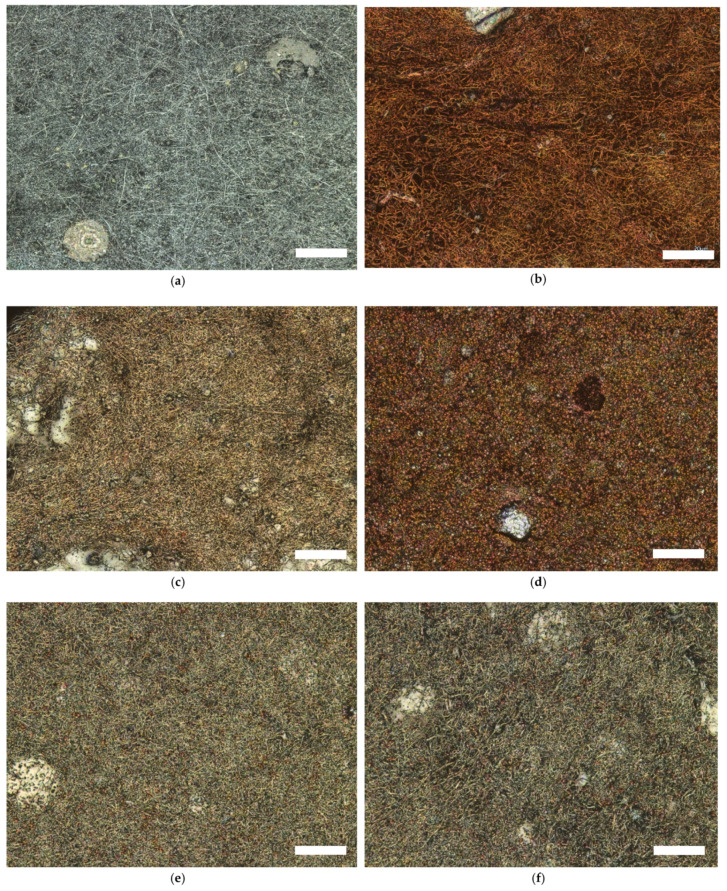
Confocal laser Scanning Microscope (CLSM) micrographs of PAN/magnetite nanofiber mats after electrospinning (**a**), after stabilization at 280 °C (**b**), after thermal treatment at 500 °C (**c**), at 600 °C (**d**), at 800 °C (**e**), and at 1000 °C (**f**). The scale bars indicate 20 μm.

The morphologies of the studied nanofiber mats are shown in the CLSM micrographs in Figure 3. The color change with the increase in temperature and the brown color is clearly recognizable (see Figure 3b–d). According to our visual observations from previous studies [58,82,83,84] the color of nanofiber mats changes from white to brown during thermal treatment and to dark brown at 800 °C.

In CLSM micrographs, the color change looks different, from light gray (Figure 3a) to brown (Figure 3b) after stabilization, and carbonized nanofiber mats (Figure 3c) look gray with the light brown again (see Figure 3e,f). Interestingly, the color brown remains visible during carbonization at 600 °C, which is atypical compared to previous studies [15,83]. The reason for this is probably that the Fe_3_O_4_ particles in the sample carbonized at 600 °C are visible, which is not the case when pure PAN is carbonized and normally represents light gray color of the surfaces of the nanofiber mat in CLSM image [15,83]. This assumption is confirmed in further CLSM images, because even at higher carbonization temperatures like 800 °C and 1000 °C, the Fe_3_O_4_ particles are still faintly visible as light brown dots. The other reason for this finding could be that, presumably, only a small part of the sample used for the CLSM studies is presented here, which is not representative of the whole sample and therefore led to these unexpected results.

For more in-depth investigations of the surface morphology and characterization of the nanofibers, PAN/magnetite mats were investigated by AFM and SEM to complement CLSM observations.

**Figure 4:**
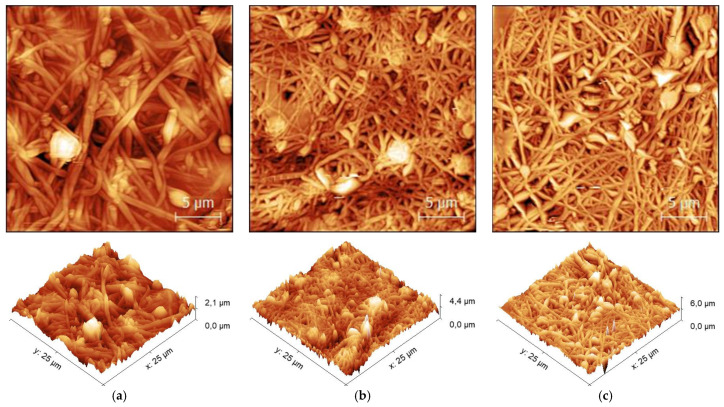
Atomic Force Microscope (AFM) micrographs of (**a**) PAN/magnetite nanofiber mat after electrospinning, (**b**) stabilized at 280 °C, and (**c**) carbonized at 600 °C. The scale bars indicate 5 μm.

Figure 4 shows AFM micrographs of magnetite nanofibers after electrospinning (Figure 4a), stabilization (Figure 4b), and carbonization at 600 °C (Figure 4c). After electrospinning, the nanofibers are relatively straight, linked together and some beads are visible. After oxidative stabilization at 280 °C (Figure 4b) and carbonization at 600 °C (Figure 4c), the nanofibers exhibit contraction. Moreover, from a visual point of view, the diameter of the nanofibers seems to decrease when increasing the temperature up to 600 °C.

**Figure 5:**
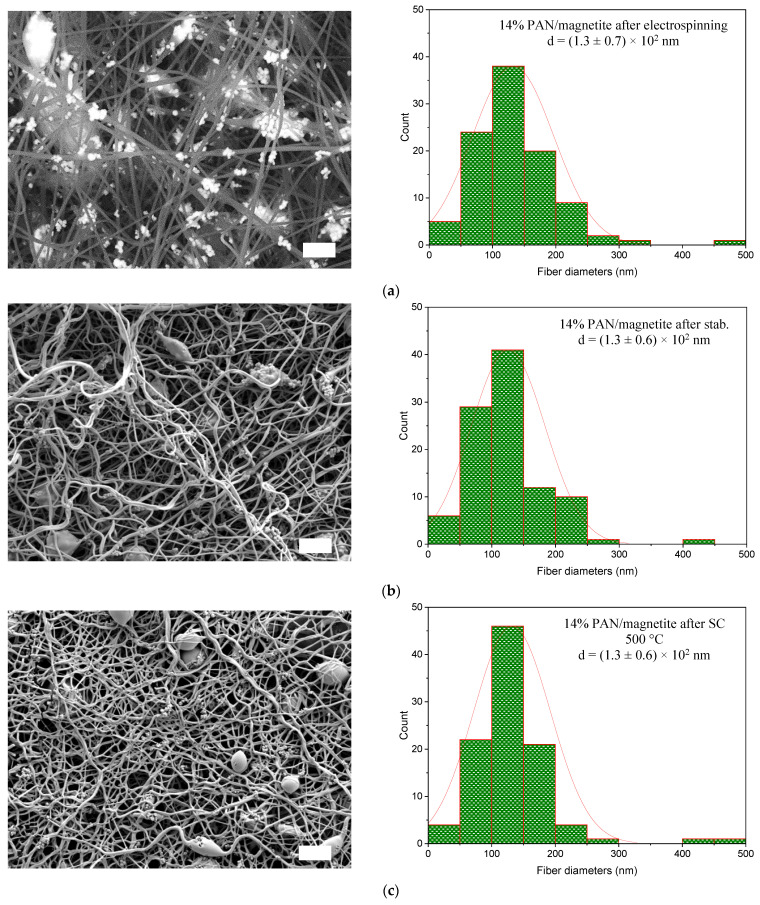

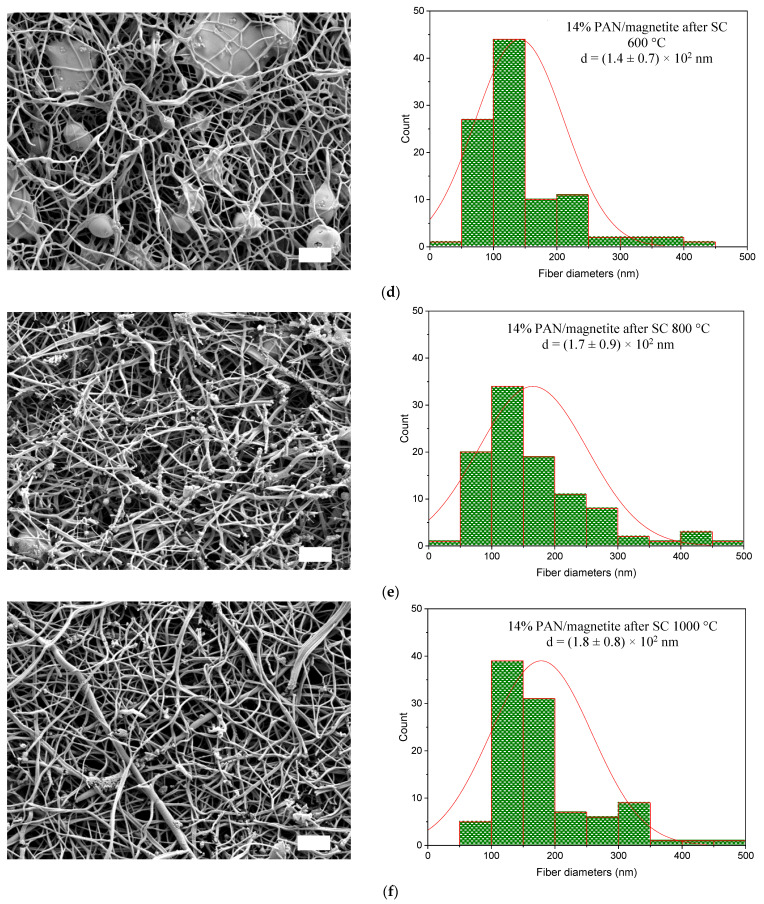
Scanning Electron Microscope (SEM) micrographs and distributions of the fiber diameters of (**a**) PAN/magnetite nanofiber mat after electrospinning, (**b**) stabilized at 280 °C, (**c**) carbonized at 500 °C, (**d**) carbonized at 600 °C, (**e**) carbonized at 800 °C, and (**f**) carbonized at 1000 °C. The scale bars indicate 2 μm.

The SEM micrographs and the graphs with nanofiber diameter in Figure 5 cannot confirm all the findings that were derived from the AFM micrographs (see Figure 5). The reason for this could be that the microscopic micrographs show only a small area of the sample and therefore do not represent the entire sample, as stated in the study by Wortmann et al. [85].

After the stabilization process, the fiber diameter decreases from (1.3 ± 0.7) × 10^2^ nm to (1.3 ± 0.6) × 10^2^, as was also suspected due to the AFM image (see Figure 4b) and corresponds to the SEM image (see Figure 5b) of the stabilized nanofiber mat.

According to Figure 5c, which shows incipient carbonization at 500 °C, fiber diameter increases almost up to the previous thermally untreated state, which was after electrospinning and exhibits (1.3 ± 0.6) × 10^2^ nm. With further increasing the carbonization temperature from 600 °C up to 1000 °C, the nanofiber diameter increased from (1.4 ± 0.7) × 10^2^ nm at 600 °C to (1.7 ± 0.9) × 10^2^ at 800 °C and finally reached (1.8 ± 0.8) × 10^2^ nm at 1000 °C (see Figure 5e,f).

By observing the surface morphology of the nanofiber mats, it was found that the nanofibers after electrospinning have relatively straight nanofibers with some beads and aggregations of magnetic particles in both AFM image (Figure 4a) and SEM image (Figure 5a). After stabilization at 280 °C, the nanofibers contract noticeably, form networks, and no longer behave as single fibers (See Figure 4b,c). With further increase of carbonization temperature from 500 °C to 600 °C, the morphology of nanofibers does not seem to change significantly and hardly any difference is seen (see Figure 4c and Figure 5c,d). It should be noted that the nanofibers continue to be more contracted in comparison to stabilized nanofiber mats (see Figure 4c and Figure 5c,d). The nanofiber mats carbonized at 800 °C (Figure 5e) and 1000 °C (Figure 5f) differ from the carbonization at the lower temperatures of 500 °C (Figure 5c) and 600 °C (Figure 5d) in that they are more contracted but show straighter fibers and exhibit some fractures, and the aggregation of nanoparticles is more visible than at the previous carbonization temperatures.

Interestingly, similarly to the study by Fokin et al. [58], the beads in the nanofiber mats almost completely disappeared at 800 °C (see Figure 5d). Furthermore, the beads are no longer visible at higher carbonization temperatures of 1000 °C (see Figure 5e). In order to preserve the beads at higher carbonization temperatures, strategies should be developed in the near future.

As known from previous studies, by adding particles or other polymers into PAN polymer, the diameter of nanofibers increases compared to pure PAN nanofibers [58,82]. Furthermore, the dimensional stability under thermal treatment is improved and the nanofiber mats contract less, are more flexible and not as fragile compared to pure PAN nanofiber mats [58,69,86].

In the study of Trabelsi et al., the PAN/magnetite nanofiber diameter after electrospinning amounted to (1.8 ± 0.5) × 10^2^ nm [21], in the study of Döpke et al. [2] to (1.2 ± 0.4) × 10^2^ nm and in the study of Fokin et al. to (1.0 ± 0.5) × 10^2^ nm [58]. The diameter of nanofibers in this study amounts to (1.3 ± 0.7) × 10^2^ nm and a significant difference between the results and other studies [2,21,58] was not observed. In general, it could be stated that the nanofiber diameter of PAN/magnetite nanofiber mats after electrospinning [2,21,58], as well as stabilization and carbonization at 500 °C and 800 °C [58], is not significantly different from previous studies. In the study by Fokin et al., the PAN/magnetite nanofiber diameter after stabilization amounted to (1.7 ± 0.7) × 10^2^ nm [58] and in this study to (0.1 ± 0.6) × 10^2^ nm and after carbonization at 500 °C amounted to (1.4 ± 0.5) × 10^2^ nm and in this study to (1.3 ± 0.6) × 10^2^ nm. Moreover, the nanofiber diameter after carbonization at 800 °C was (2.0 ± 0.9) × 10^2^ nm in the case of Fokin et al. [58] and (1.8 ± 0.8) × 10^2^ nm in this study. However, it should also be mentioned that these differences in nanofiber diameter are not significant statistically due to the wide dispersion of the measured fiber diameters. In addition, the arbitrary choice of the investigated sample areas also partly plays a role.

**Figure 6:**
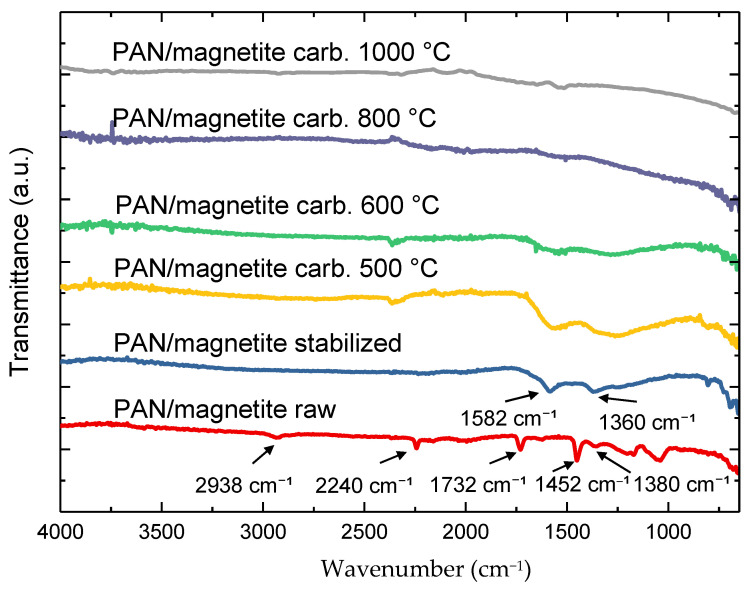
Fourier-transform infrared spectroscopy (FTIR) measurements of PAN/magnetite samples after electrospinning, stabilization, and carbonization at 500 °C, 600 °C, 800 °C, and 1000 °C. The lines are vertically offset for clarity.

FTIR measurements were performed on the PAN/magnetite nanofiber mats after electrospinning, stabilization, and carbonization at 500 °C, 600 °C, 800 °C, and 1000 °C (see Figure 6).

The comparison of the spectra shows that the transmittance of PAN/magnetite mats consists of the typical PAN peaks. Significant changes occur during the stabilization and carbonization processes and almost all the peaks disappear at carbonization at 800 °C or higher. Below 800 °C, according to our previous study [58], carbonization is not complete, and the peaks are visible.

The stretching vibrations of the C≡N nitrile functional group at 2240 cm^−1^ and the carbonyl (C=O) stretching peak at 1732 cm^−1^ can be observed. In addition, the bending and stretching vibrations of CH_2_ at 2938 cm^−1^, 1452 cm^−1^, and 1380 cm^−1^ can be identified. This characterization of the nanofiber mats is well-known from previous studies [84,85,86].

In FTIR spectra of the stabilized nanofiber mats, the most prominent peaks are those of C=N stretching vibrations at 1582 cm^−1^, C=C stretching vibration at 1660 cm^−1^, as well C-H bending and C-H_2_ wagging around 1360 cm^−1^ [58].

As found in a previous study, the temperature treatment at 500 °C is not sufficient to achieve complete carbonization and leads to incipient carbonization [87]. This is confirmed in the FTIR spectrum, which still looks very similar to the stabilized sample (see Figure 6). After carbonization at 600 °C, the peaks disappeared almost completely and when carbonized at 800 °C and 1000 °C, only a few functional groups remained, resulting in the typical high absorption of carbon. As can be seen in the FTIR, the complete carbonization occurs at 800 °C, as known from our previous studies [82,83,86]. Moreover, similarly to our previous study [58], when comparing the FTIR measurements with those on pure PAN nanofiber mats or PAN mixtures with TiO_2_, gelatin, etc., no significant difference could be detected due to the embedded magnetic nanoparticles [58,82,86].

Carbonization at high temperatures above 800 °C is challenging because as the temperature increases, the nanofiber morphology also changes due to the incited chemical processes. At this point, defined surface morphology should be weighed up against the applied carbonization temperature. From previous studies it is known that the nanofiber mats are relatively flexible after electrospinning; after stabilization and carbonization, however, they lose flexibility either partially or completely and become very fragile at carbonization higher than 800 °C [15,58,82,83]. Therefore, the carbonization temperature of 1000 °C was relatively challenging for PAN/magnetite mats and confirmed the assumption that the desired surface morphology and carbonization temperature should be considered to obtain desirable magnetic carbon nanofibers for defined applications. As can be seen in FTIR, after carbonization at 1000 °C, almost all the functional groups have disappeared and become the similar structure of graphite fibers [88].

It is known from literature that the conductivity increases with increasing carbonization temperature [89,90]. The study by Liu et al. confirms the increase in the conductivity of carbon nanofibers prepared from PAN precursor with increasing temperature. At 400 °C, the conductivity of the carbon fibers has reached 0.015 S-cm^−1^ and at 700 °C, 0.018 S-cm^−1^. Moreover, the conductivity increases significantly with carbonization at 1000 °C and attains 105.44 S-cm^−1^ [90].

There are various reasons for the increase in conductivity. On the one hand, the fiber diameter decreases due to the prevailing chemical processes in the heat treatment process. On the other hand, the preferred orientation of the carbon layer with a flat structure increases significantly, while the void volume fraction and the crystallite size increase significantly [90]. During the carbonization process, various processes such as oxidation, aromatization, cyclization, dehydrogenation, and crosslinking occurred [91,92,93]. The increase in temperature leads to the coalescence of the flake structures, which are oriented along the longitudinal direction, and this finally leads to a significant increase in conductivity [90,92]. For this reason, carbon nanofibers carbonized at 1000 °C showed a sufficient degree of carbonization and structural integrity [90].

**Figure 7:**
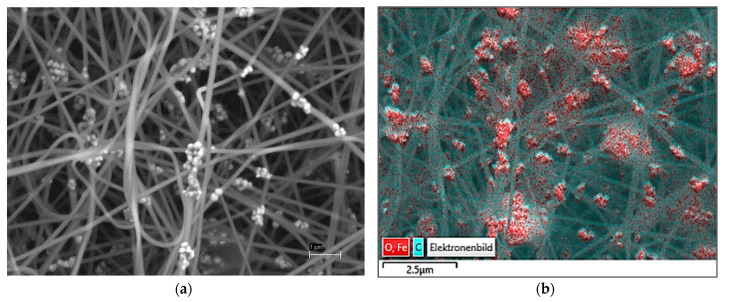
Scanning Electron Microscope (SEM) image of PAN/magnetite nanofiber mat (**a**) and Energy Dispersive X-Ray Spectroscopy (EDS) spectrum showing magnetite in red color (**b**). The scale bars indicate 1 μm (**a**) and 2.5 μm (**b**).

To investigate the distribution of magnetite in the samples in more detail, SEM image and EDS mapping were used. When comparing the SEM image (Figure 7a) and the EDS map (Figure 7b), it is suggested that the highest particle concentration is located in the beads.

**Figure 8:**
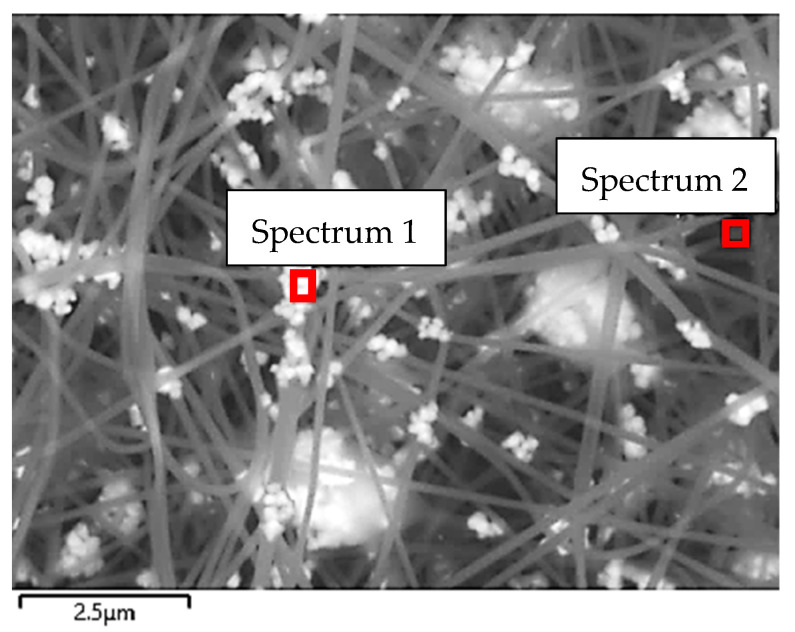
SEM image of PAN/magnetite nanofiber mat (**a**) spot on a bead (Spectrum 1); and (**b**) spot on a nanofiber (Spectrum 2). The scale bar indicate 2.5 μm.

Figure 8 shows the surface scanned with EDS for a more detailed examination and the spectra of the materials inside this surface. As can be seen in the SEM image and marked with red frames, the EDS spectrum was recorded in a microscopic area of the sample (see Figure 8).

**Figure 9:**
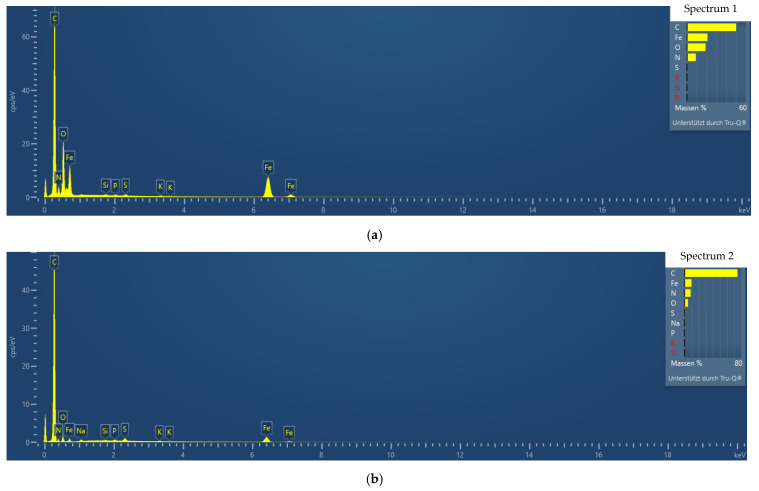
EDS spectra of PAN/magnetite nanofiber mat (**a**) spot on a bead; and (**b**) spot on a nanofiber.

The examined areas were marked with a red frame (see Figure 8) and the EDS spectra were generated for these areas (see Figure 9a,b). The first EDS (Spectrum 1) defines a small area where a bead is located, and the second EDS (Spectrum 2) was directed to nanofiber.

It could be observed that carbon (C) is present in both EDS spectra in a large amount at 0.3 keV. The peaks at 0.7 keV, 6.4 keV, and 7.1 keV in both EDS spectra (see Figure 9a,b) can be identified as being related to iron (Fe). Looking at the EDS spectra 1 (Figure 9a), it is noticeable that the iron peak (Fe) is higher in the EDS spectrum 1 directed at the bead compared to the EDS spectrum 2 directed at the nanofiber. In addition, the EDS spectrum 1 shows strong peaks of the element iron (Fe), confirming that the magnetite nanoparticles are agglomerated within the beads. Similar to our previous study [21], larger amounts of magnetite can be suspected in beads than are expected to be present in nanofibers.

## 4. Conclusions

In this study, PAN/magnetite nanofiber mats were prepared by adding nanoparticles into a polymer solution used for needleless electrospinning method. Oxidative stabilization and carbonization at 500 °C, 600 °C, 800 °C, and 1000 °C were performed, and the resulting surface morphologies of the nanofiber mats were studied and discussed. According to this study, the addition of nanoparticles increased the fiber average and improved the dimensional stability. The agglomerations of magnetite nanoparticles were largely detected in beads by means of SEM and EDS spectra. Similar to our previous study [17], larger amounts of magnetite are suspected in the beads than in the nanofibers. The addition of nanoparticles changes the morphology of nanofiber mats. New types of nanofiber mats can be manufactured from numerous polymers and different raw materials, which can lead to defined functions and different sensor capacities and still allow for much innovation in the field of sensor technology.

Carbonization of nanofiber mats at 1000 °C is challenging because, due to the incited chemical processes, the surface morphology changes. The conductivity at high temperatures increases, which is beneficial for the speed of signal transmission when such magnetic carbon nanofiber mats will be used for the construction of novel single photon avalanche diodes (SPADs), where these properties are advantageous. The excellent mechanical properties as well as the large magnetization and shielding against electromagnetic interference can reduce the noise in the (SPAD) sensor and increase the speed of signal transmission. This present work aims to provide the basis for this novel single photon avalanche diode (SPAD) construction design.

## Data Availability

The data created in this study are fully depicted in the article.
